# Immunization of Pigs by DNA Prime and Recombinant Vaccinia Virus Boost To Identify and Rank African Swine Fever Virus Immunogenic and Protective Proteins

**DOI:** 10.1128/JVI.02219-17

**Published:** 2018-03-28

**Authors:** James K. Jancovich, Dave Chapman, Debra T. Hansen, Mark D. Robida, Andrey Loskutov, Felicia Craciunescu, Alex Borovkov, Karen Kibler, Lynnette Goatley, Katherine King, Christopher L. Netherton, Geraldine Taylor, Bertram Jacobs, Kathryn Sykes, Linda K. Dixon

**Affiliations:** a Biodesign Institute, Arizona State University, Tempe, Arizona, USA; b The Pirbright Institute, Pirbright, Woking, Surrey, United Kingdom; University of Southern California

**Keywords:** African swine fever virus, DNA vaccines, immune markers, vaccinia virus, veterinary vaccine development

## Abstract

African swine fever virus (ASFV) causes an acute hemorrhagic fever in domestic pigs, with high socioeconomic impact. No vaccine is available, limiting options for control. Although live attenuated ASFV can induce up to 100% protection against lethal challenge, little is known of the antigens which induce this protective response. To identify additional ASFV immunogenic and potentially protective antigens, we cloned 47 viral genes in individual plasmids for gene vaccination and in recombinant vaccinia viruses. These antigens were selected to include proteins with different functions and timing of expression. Pools of up to 22 antigens were delivered by DNA prime and recombinant vaccinia virus boost to groups of pigs. Responses of immune lymphocytes from pigs to individual recombinant proteins and to ASFV were measured by interferon gamma enzyme-linked immunosorbent spot (ELISpot) assays to identify a subset of the antigens that consistently induced the highest responses. All 47 antigens were then delivered to pigs by DNA prime and recombinant vaccinia virus boost, and pigs were challenged with a lethal dose of ASFV isolate Georgia 2007/1. Although pigs developed clinical and pathological signs consistent with acute ASFV, viral genome levels were significantly reduced in blood and several lymph tissues in those pigs immunized with vectors expressing ASFV antigens compared with the levels in control pigs.

**IMPORTANCE** The lack of a vaccine limits the options to control African swine fever. Advances have been made in the development of genetically modified live attenuated ASFV that can induce protection against challenge. However, there may be safety issues relating to the use of these in the field. There is little information about ASFV antigens that can induce a protective immune response against challenge. We carried out a large screen of 30% of ASFV antigens by delivering individual genes in different pools to pigs by DNA immunization prime and recombinant vaccinia virus boost. The responses in immunized pigs to these individual antigens were compared to identify the most immunogenic. Lethal challenge of pigs immunized with a pool of antigens resulted in reduced levels of virus in blood and lymph tissues compared to those in pigs immunized with control vectors. Novel immunogenic ASFV proteins have been identified for further testing as vaccine candidates.

## INTRODUCTION

African swine fever virus (ASFV) causes an acute hemorrhagic fever, African swine fever (ASF), with high mortality in domestic pigs and which has severe socioeconomic consequences in affected countries. Currently the disease is endemic to or causes sporadic outbreaks in most sub-Saharan African countries. Outside Africa, ASF has been enzootic in Sardinia since 1978. Following the introduction of ASF to Georgia in 2007, the disease spread to Eastern Europe, including the Russian Federation, Belarus, Ukraine, Lithuania, Poland, Latvia, Estonia, and Moldova (OIE World Animal Health Information System; http://www.oie.int/wahis_2/public/wahid.php/Wahidhome/Home). The lack of a commercially available vaccine limits disease control options to reliance on rapid detection and implementation of quarantine and slaughter. The complexity of the virus, which encodes 150 to 167 proteins, contributes to the difficulty in obtaining an effective vaccine.

It is well established that pigs that recover from infection with attenuated ASFV isolates can be protected against challenge with related virulent viruses (1–4). Immunization of pigs with attenuated strain OURT88/3 induces protection against lethal challenge that is dependent on CD8^+^ cells, since protection was abrogated by depletion of this cell subset (5). In pigs, several different subclasses of CD8^+^ cells have been defined. These include CD8 singly positive cells and CD4 CD8 doubly positive subsets which are perforin positive and have swine leukocyte antigen I (SLA I)-restricted cytotoxic activity. In addition, CD8 is also expressed on additional cell subsets, including NK cells (6). Although the depletion studies did not provide information on which CD8^+^ cell subset was important for protection, additional studies have shown a correlation between induction of the CD4^+^ CD8^+^, perforin^+^ cytotoxic T lymphocytes (CTL) with protection induced by the attenuated OURT88/3 strain (6). However, protection induced by the attenuated strain NHP/68 has been correlated with high levels of NK cells (4). A role for antibodies is also indicated, since passive transfer of antibodies from immune to naive pigs can induce partial protection (7).

Although live attenuated ASFV strains can induce protection of up to 100%, they can cause adverse reactions and may have other safety issues. Vaccines which achieve protection by delivery of one or more virus antigens would have advantages in terms of safety, but knowledge of the protective antigens is required. Attempts to induce protection against lethal ASFV challenge by immunization of pigs with recombinant proteins or by DNA vaccination have had partial success. In one study, recombinant proteins p54 and p72 induced partial protection against lethal challenge, although in another study no protection was observed with these proteins despite the induction of neutralizing antibodies (8–10). Recombinant ASFV hemagglutinin (HA) or CD2v protein (EP402R gene) has also induced partial protection of pigs (11). Partial protection of 30 to 50% of immunized pigs has also been achieved by DNA vaccination with a fusion of the p54 and p30 proteins to the extracellular domain of the CD2v protein or by immunization with an expression library (12–14). However, other ASFV proteins are likely to have the potential to be protective but have not been systematically investigated.

In the current study, we carried out a screen of 47 ASFV-encoded antigens to determine which induced cellular responses and to assess their potential for inducing protection. These were selected to include proteins with different functions and timing of expression. To achieve this, we constructed 3 different ASFV libraries, a gene library for DNA vaccination (15–17), a recombinant vaccinia virus (rVACV) library (18–20), and a library for expression and capture of individual recombinant proteins using an *Escherichia coli in vitro* transcription and translation system. We immunized pigs with pools of these ASFV antigens delivered by a DNA prime and recombinant vaccinia virus boost and ranked the immune responses to the individual captured proteins. Pigs immunized with this pool of antigens had lower levels of virus in blood, tonsil, spleen, and submandibular lymph node than those immunized with control antigens following challenge with a lethal dose of ASFV.

## RESULTS

### Delivery of ASFV gene pools by DNA prime and recombinant vaccinia virus boost to rank immune responses in Babraham pigs (experiment 1).

ASFV antigens were selected to represent proteins of different functions, timing of expression, and predicted cellular localization. These functional classes included enzymes involved in virus genome replication and transcription, proteins with roles in immune evasion, virion structural proteins, membrane proteins, and proteins of unknown function. The larger proteins were cloned in several fragments to facilitate their expression. This approach was taken to represent the diversity of antigens expressed during an ASFV infection. These 47 genes or gene fragments were cloned in vectors for gene immunization, for expression in recombinant vaccinia virus vectors, and for recombinant protein expression by *in vitro* transcription and translation (see Materials and Methods). In the first experiment, inbred Babraham pigs were immunized to compare and rank the responses to individual antigens. Four groups of pigs were immunized by DNA prime and rVACV boost as described in Materials and Methods, with 4 weeks between the final DNA vaccination and rVACV boost. Group 1 contained 6 pigs immunized with a set of 20 ASFV antigens comprising different functional classes (labeled set 1 [Table 1]) plus p30 (CP204L) and VP72 (B646L), two well-characterized immunogenic ASFV proteins. Group 2 contained 6 pigs immunized with another set of 20 ASFV antigens of different functional classes (labeled set 2 [Table 1]) plus p30 (CP204L) and VP72 (B646L). Group 3 contained 5 pigs immunized with 10 ASFV antigens which are known or predicted to be present on the surface of the intracellular mature or extracellular ASFV virion particles (Table 1), in addition to p30 (CP204L) and VP72 (B646L). These were selected as proteins likely to be important for induction of antibody responses. Group 4 contained 5 pigs and these were immunized with p30 (CP204L) and VP72 (B646L) alone (Table 1). The reason for including the p30 (CP204L) and VP72 (B646L) antigens in each group was to determine if the size and composition of the antigen pool influenced the immune response to these proteins.

**TABLE 1:**
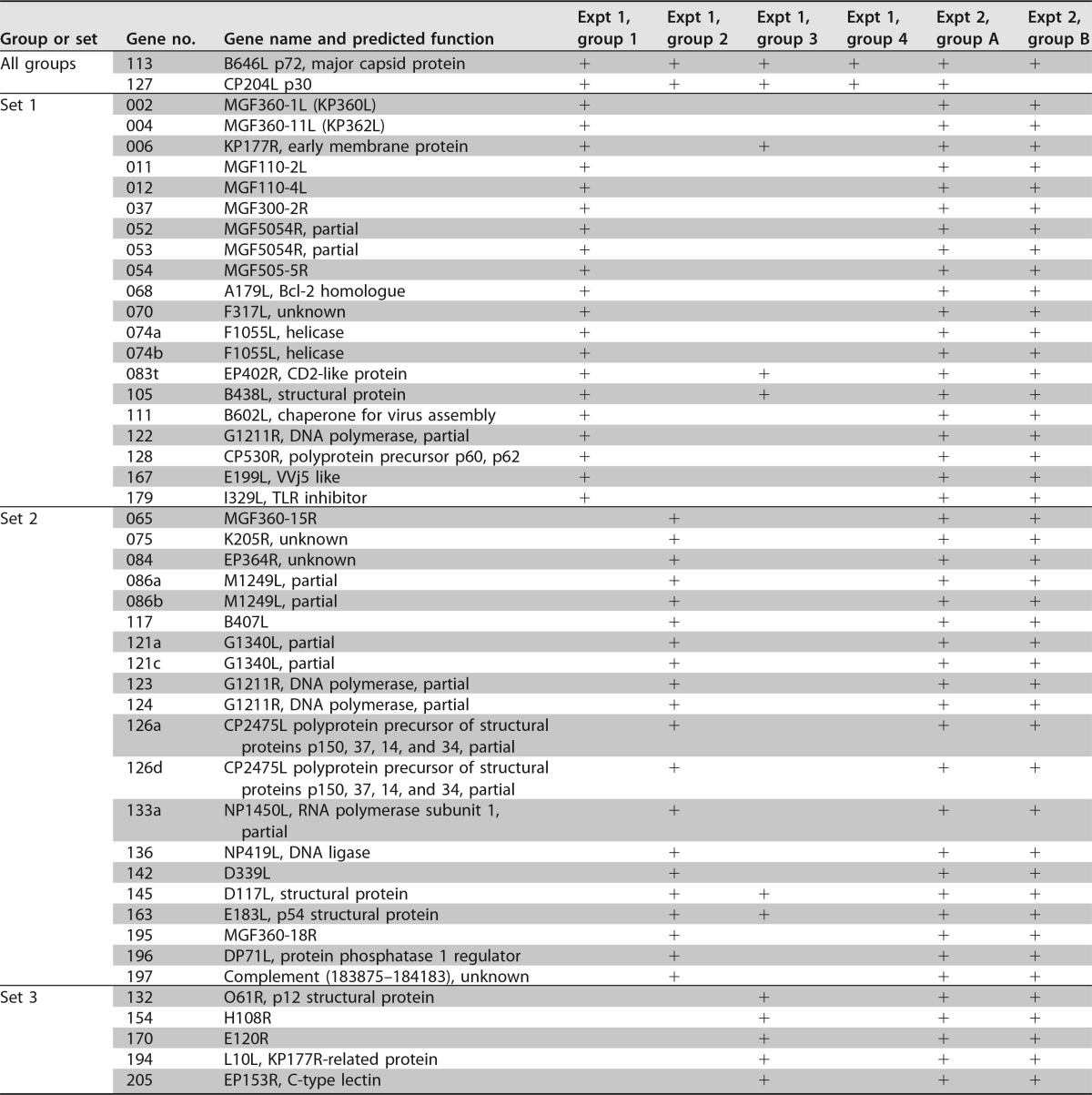
Antigens tested by DNA prime and recombinant vaccinia virus boost in pigs^*a*^

a The antigens selected for testing by immunization of pigs are indicated, with the relevant antigen set in the first column, an antigen number in the second column, and the gene name and predicted function in the third column. The remaining columns show the antigens that were present (“+”) in the different groups in the immunization experiment (experiment 1; groups 1 to 4) and in the immunization and challenge experiment (experiment 2; groups A and B).

**FIG 1:**
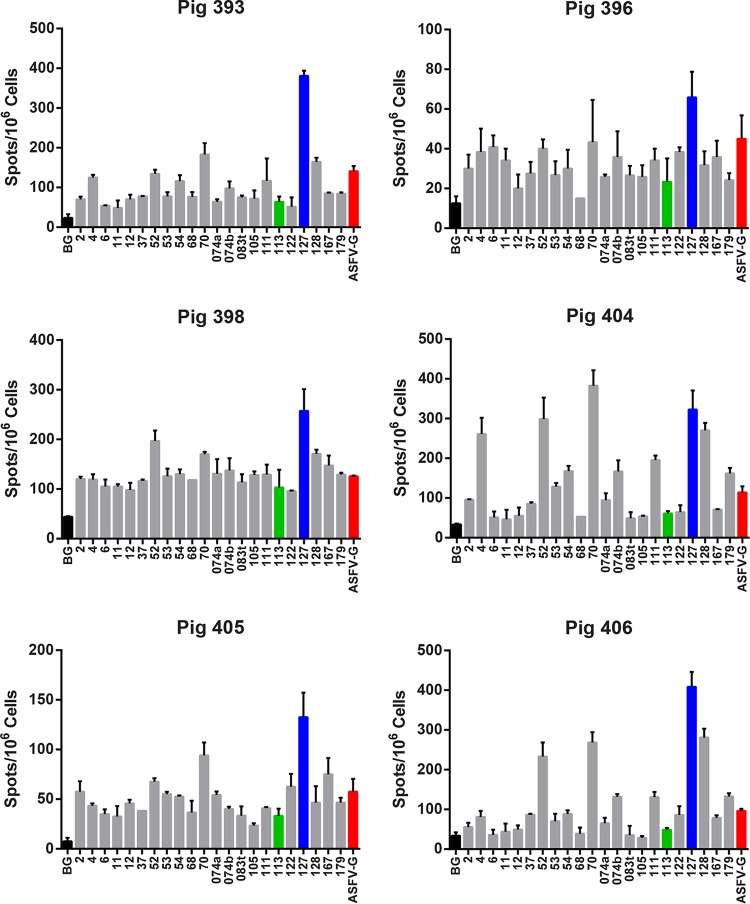
Interferon gamma ELISpot assay results for group 1 antigens. Six Babraham pigs were immunized with a pool of 22 DNA plasmids and boosted 4 weeks later with rVACV expressing individual recombinant antigens as shown in Table 1, group 1. PBMCs purified 4 weeks postboost were stimulated overnight with the indicated antigens, medium alone (BG), or the Georgia 2007/1 ASFV isolate (ASFV-G). The IFN-γ response was determined by ELISpot assay, and each graph shows the results for one pig. Error bars indicate the SDs from the means of duplicate wells. The black, green, blue, and red bars highlight the response to medium alone, antigen 113 (B646L/p72), antigen 127 (CP204L/p30), and Georgia 2007/1, respectively.

Cellular immune responses to the antigens were analyzed by stimulating peripheral blood mononuclear cells (PBMCs) from immunized pigs with recombinant individual ASFV proteins produced by *in vitro* transcription and translation or with ASFV isolate Georgia 2007/1. Responses were measured as numbers of interferon gamma (IFN-γ)-producing cells by enzyme-linked immunosorbent spot (ELISpot) assays. Figure 1 shows results from IFN-γ ELISpot assays performed with lymphocytes from the 6 pigs in group 1 immunized with the 20 antigens of set 1. The levels of response in different pigs after stimulation with whole ASFV Georgia 2007/1 virus varied between 37 and 132 IFN-γ-producing cells per million cells (Fig. 1), and the background response to medium alone varied between 13 and 35% of this value (Fig. 1). A number of individual antigens induced a greater number of IFN-γ-producing cells than whole virus, and the antigens induced a response at least two-thirds of that induced by Georgia 2007/1 in at least four of the six pigs. Antigen 127 (p30) induced the greatest number of IFN-γ-producing cells from 5 of the 6 pigs in group 1, varying from 145 to 422% relative to the number induced by ASFV Georgia 2007/1, taken as 100% (Fig. 1). The response for antigen 070 (F317L) varied from 96 to 334% of the response to whole virus. Antigen 052 (MGF505-4R) induced from 89 to 261% of the response observed following stimulation of cells with whole virus. Eight antigens, 004 (MGF360-11L), 052 (MGF505-4R), 070 (F317L), 074b (F1055L), 111 (B602L), 128 (CP530R), 167 (E199L), and 127 (CP204L/p30), induced responses greater than 70% of that induced by ASFV Georgia 2007/1 in all 6 pigs. A further 4 antigens, 002 (MGF 360-1L), 053 (MGF505-4R), 054 (MGF505-5R), and 122 (G1211R), induced a level of response that was 70% that of the Georgia 2007/1 isolate in cells from 3 or more of the pigs.

**FIG 2:**
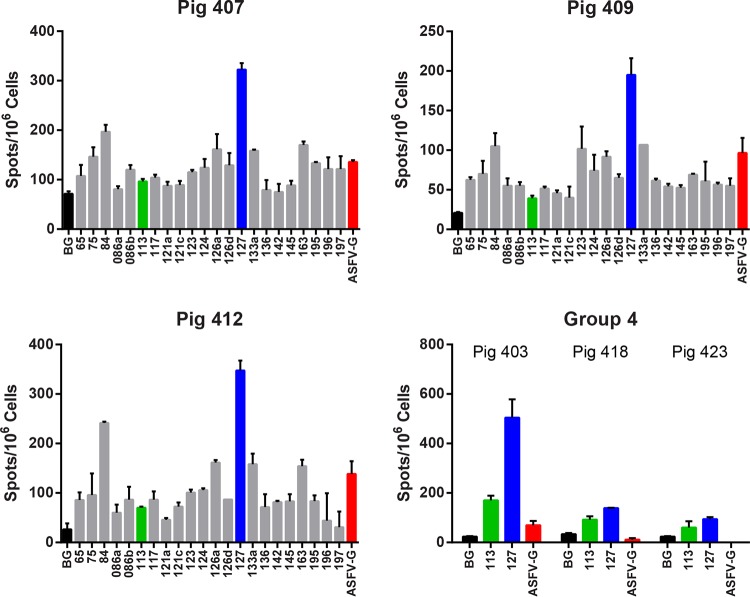
Interferon gamma ELISpot assay results for group 2 and group 4 antigens. Babraham pigs were immunized with pools of DNA plasmids and boosted 4 weeks later with rVACV expressing an individual recombinant antigen as shown in Table 1. Numbers of IFN-γ-producing cells were measured by ELISpot assay in 3 (407, 409, and 412) of the 5 pigs immunized with 22 antigens as shown in Table 1, group 2. These included antigens 113 and 127, which were included in all groups. ELISpot assay results are shown from 3 pigs (403, 418, and 423) of the 5 pigs immunized with antigens 113 and 127 alone (see Table 1, group 4). PBMCs purified 4 weeks after the boost with rVACV were stimulated overnight with the indicated antigens, medium alone (BG), or the Georgia 2007/1 ASFV isolate (ASFV-G). The IFN-γ response was determined by ELISpot assay, and error bars indicate the SDs from the means of duplicate wells. The black, green, blue, and red bars highlight the response to medium alone, antigen 113 (B646L/p72), antigen 127 (CP204L/p30), and Georgia 2007/1, respectively. PBMCs from some pigs (397, 411, and 424 in group 2 and 417 and 421 from group 4) failed to respond to stimulation by antigens or virus, and results from these pigs are not shown. This may be due to experimental error in storage or recovery of cells after freezing.

For group 2 immunized with the 20 antigens of set 2 (Table 1), the IFN-γ responses of cells from 3 pigs to the individual antigens were analyzed (Fig. 2); antigens which induced a response at least two-thirds of that induced by whole virus in all three pigs are shown in Fig. 2. Antigen 127 (p30) induced the highest response in all 3 pigs, ranging from 201 to 249% of that induced by ASFV Georgia 2007/1. Antigen 084 (EP364R) also induced high responses in all pigs, ranging from 108 to 174% that of ASFV Georgia 2007/1. Seven antigens, 084 (EP364R), 123 (G1211R), 124 (G1211R), 126a (CP2475L), 133a (NP1450L partial), 163 (E183L), and 127 (CP204L/p30), induced responses greater than 70% that of ASFV Georgia 2007/1 in all three pigs (Fig. 2).

**FIG 3:**
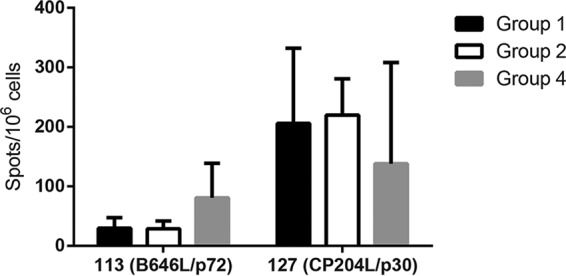
Intergroup comparison of responses to antigens 113 and 127. The graph shows the means and SDs of the IFN-γ responses from groups 1, 2, and 4 to antigen 113 (B646L/p72) and antigen 127 (CP204L/p30).

Cellular immune responses in pigs from groups 1, 2, and 4 to antigens 127 (CP204L/p30) and 113 (B646L/p72) were compared, using IFN-γ ELISpot assays, to evaluate if the responses varied when antigen sets varied in content and size. As expected, cells from pigs in group 4 immunized with antigens 127 (CP204L/p30) and 113 (B646L/p72) alone responded less well to whole virus than cells from pigs in groups 1 and 2 that were immunized with antigen sets of 20 plus 127 and 113. After the response to medium alone was subtracted, the responses to antigens 113 and 127 in group 4 varied between 37 and 147 and between 34 and 334 spots per million cells, respectively. The responses to the two antigens in the three different groups were not significantly different (one-way analysis of variance [ANOVA]) (Fig. 3). This showed that differences in the composition or the size of the pool did not affect the cellular immune responses to the well-characterized ASFV antigens CP204L/p30 and B646L/p72.

**FIG 4:**
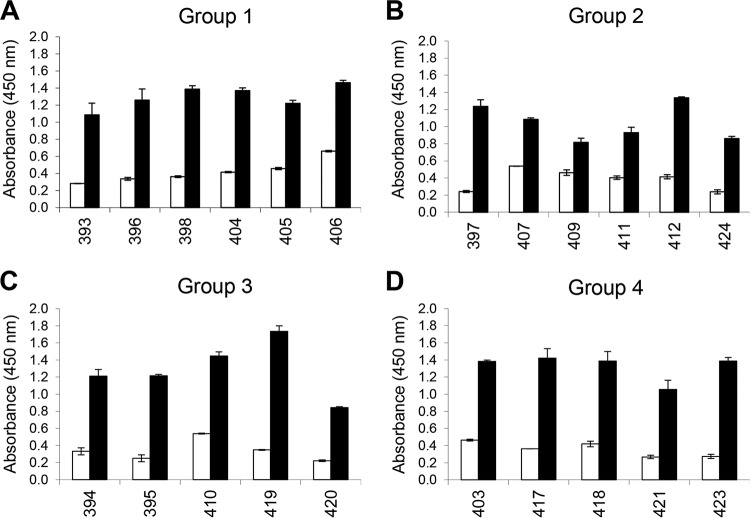
Antibody response to p30 in pigs from groups 1, 2, 3, and 4. Serum antibody responses to recombinant protein p30 were measured by ELISA in pigs (*x* axis shows the pig number) immunized with antigens shown in Table 1, group 1 (A), group 2 (B), group 3 (C), and group 4 (D). Open bars indicate results from prebleeds collected prior to immunization, and closed bars indicate final bleeds collected at termination of the experiment. Error bars show the SDs from duplicate measurements. Results are shown for all of the immunized pigs (group 1, *n* = 6; group 2, *n* = 6; group 3, *n* = 5; and group 4, *n* = 5).

**FIG 5:**
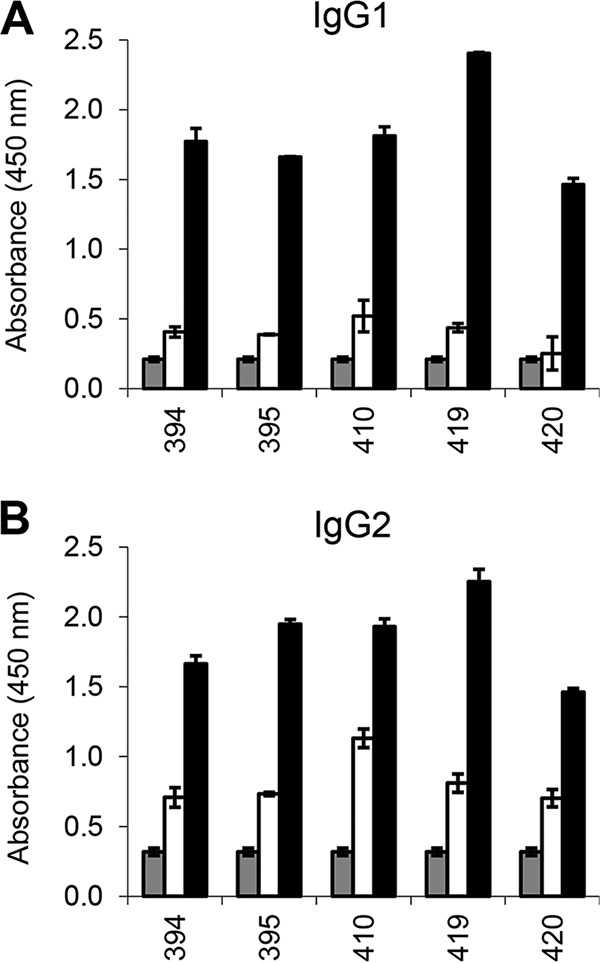
IgG1 and IgG2 responses to p30 in immunized pigs from group 3. Serum IgG1 (A) and IgG2 (B) responses to recombinant protein p30 were measured by ELISA in all pigs (*n* = 5) from group 3. The *x* axis shows the pig number. Gray bars indicate absence of added serum, open bars indicate prebleeds prior to immunization, and closed bars indicate final bleeds collected at termination. Error bars show SDs from duplicate measurements.

**FIG 6:**
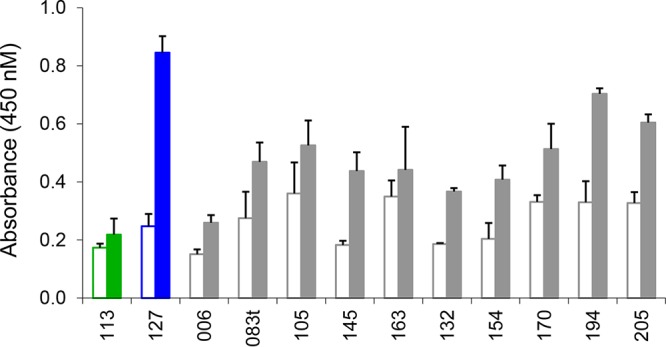
Antibody response to individual antigens from group 3. Antibody responses to each of the 12 individual antigens (see Table 1, group 3) are shown on the *x* axis as measured by ELISA in sera from pig 395 from group 3. Open bars indicate prebleeds, and closed bars indicate final bleeds. Error bars show SDs from duplicate measurements. Serum from the other 4 pigs immunized in group 3 were not analyzed.

The antibody response to antigen 127 (CP204L/p30) was analyzed by enzyme-linked immunosorbent assay (ELISA) using sera from the immunized pigs in groups 1 to 4 (Fig. 4). The results showed that responses to p30 were consistently high in all pigs from all groups and therefore that the size of the antigen pool had not affected antibody responses to this individual protein. Analysis of the isotype of antibodies induced identified both IgG1 and IgG2 antibodies, indicating a broad isotype spectrum (Fig. 5). Antibody responses to individual antigens in group 3 were compared by ELISA for one of the pigs (pig 395 [Fig. 6]). This showed that following immunization, antibody responses to individual antigens varied. Responses to antigen 127 (CP204L/p30) were highest and were approximately 3- to 4-fold above background levels. Other antigens showing a >2-fold increase in response included antigens 194 (L10L), 145 (D117L), and 205 (EP153R). Neutralizing antibodies were not detected in sera from any of the pigs (data not shown).

### Immunization of pigs with a pool of 47 ASFV genes and effect on ASFV challenge (experiment 2).

The experiment described above indicated that good cellular and antibody responses had been induced in pigs following immunization by a DNA prime/rVACV boost and that delivery of the antigens in larger pools had not caused a great reduction in response to individual antigens. We therefore carried out a second experiment in which pigs were challenged with a lethal dose of ASFV after the DNA prime and rVACV boost immunization. The previous experiments had shown that the p30 protein was very immunogenic and induced strong antibody and cellular responses. We considered it possible that including this antigen may direct the immune responses to p30 rather than more broadly to other potentially protective antigens. Therefore, we immunized pigs with pools of DNA and rVACV expressing ASFV antigens containing or lacking the p30 protein.

**FIG 7:**
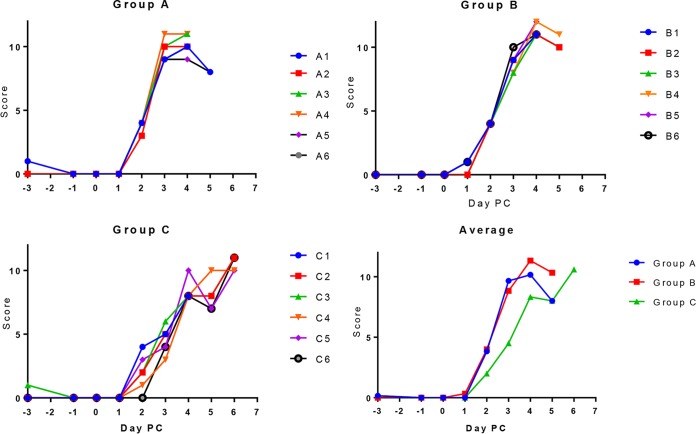
Clinical scores from pigs immunized with different pools of antigens following lethal challenge with virulent ASFV Georgia 2007/1. Three groups of 6 Babraham pigs were immunized with pools of DNA plasmids twice, 2 weeks apart. Each immunization contained 10 μg of DNA total and CpG adjuvant. Animals were boosted after a further 3 weeks and 5 weeks with rVACVs expressing antigens as shown in Table 1, experiment 2 (groups A and B). Group A consisted of all 47 antigens and group B all except CP204L/p30. Group C contained irrelevant control antigens gp160, AAT, and HA. Three weeks after the final rVACV boost, pigs were challenged with 10^4^ HAD_50_ of virulent Georgia 2007/1 isolate by the intramuscular route. Clinical scores (*y* axis) were recorded at different days postchallenge (PC; *x* axis). The results are shown for individual pigs in each group and for the average for each group.

Three groups of 6 Babraham pigs were immunized. Group A pigs were immunized with DNA and rVACV expressing all of the previously analyzed 47 ASFV antigens (Table 1), group B with all ASFV antigens except for that encoding CP204L/p30 (Table 1), and group C only with control antigens gp160, α1-antitrypsin (AAT), and HA (see Materials and Methods). The regimen for immunization was a double prime with DNA plasmids (10 μg total) and CpG adjuvant and 2 weeks later a boost with DNA (10 μg total) and CpG adjuvant. Pigs were boosted with rVACVs 3 weeks later, with a second boost 2 weeks after that. Three weeks after the final rVACV boost, pigs were challenged intramuscularly with 10^4^ 50% hemadsorbtion doses (HAD_50_) of ASFV Georgia 2007/1 and the development of clinical signs was recorded. Blood samples were collected at day 0, at 3 days postchallenge, and at termination for measurement of virus load. Tissue samples were collected postmortem. Pigs in all 3 groups developed clinical signs on day 3 postchallenge and were euthanized on day 5 or 6 when they reached the moderate-severity humane endpoint for the experiment (Fig. 7). Statistical analysis by 2-way ANOVA of mean clinical scores per group showed that scores were significantly higher in groups A and B than in control group C (*P* = 0.0001) on days 2, 3, and 4 postchallenge. This suggests a possible immune enhancement of disease in those pigs immunized with vectors expressing ASFV antigens, although the mechanism is unknown.

Postmortem examination showed that all pigs had signs typical of the early stages of acute ASF disease, including signs of hemorrhage and enlargement of the spleen and lymph nodes.

**FIG 8:**
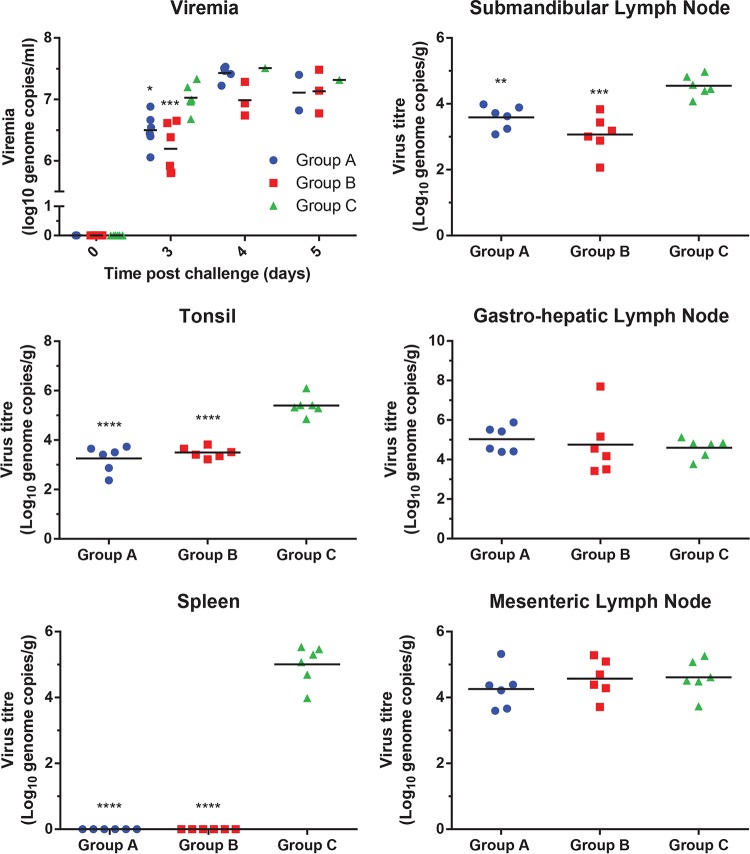
ASFV genome in blood and tissue samples collected from different pigs. Levels of ASFV DNA detected in samples collected from pigs immunized with different groups of ASFV antigens (Table 1, groups A and B) or with unrelated group C antigens (control) are shown as log 10 genome copies per milliliter of blood or gram of tissue (*y* axis). Each panel shows results for viremia at different days postchallenge or different tissues collected at termination as indicated. Results for individual pigs are shown. Black lines indicate the means of each group, and asterisks indicate significant differences between group C and either group A or group B (ANOVA; *, *P* ≤ 0.05; **, *P* ≤ 0.01, ***, *P* ≤ 0.001; ****, *P* ≤ 0.0001).

The virus genome load in tissues and blood was measured using quantitative PCR (qPCR) (Fig. 8). This showed a significant reduction (one-way ANOVA) in virus load in blood at day 3 postchallenge and in the spleen, tonsils, and submandibular lymph nodes in pigs in groups A and B, which were immunized with pools of DNA and rVACVs expressing ASFV antigens, compared to virus loads in pigs in group C, immunized with the control antigens. In fact, virus loads were 10- to 100-fold lower in the submandibular lymph nodes, tonsils, and blood of the pigs in groups A and B than in the controls, and virus loads were reduced to undetectable levels in the spleens of group A and B. However, in the gastrohepatic and mesenteric lymph nodes, similar levels of virus genome were detected in samples from all three groups of pigs (Fig. 7). These results indicate that clearing of virus had occurred in some tissues and blood by the day the pigs were killed.

## DISCUSSION

In this study, we aimed to identify immunogenic and potentially protective antigens encoded by ASFV for future investigation as targets for vaccine development. In addition, the identification of immunogenic proteins would provide additional biomarkers for serological diagnosis. The development of a safe and efficacious vaccine for ASFV would have a high economic impact since currently no vaccine is available and disease control relies on implementation of quarantine and mass slaughter of exposed pigs.

Previously, expression library immunization has been used to identify immunogenic and candidate protective antigens encoded by genomes of complex pathogens. This approach has several advantages: it does not require prior knowledge of the protective antigens, and it may identify potentially protective antigens that either are not exposed or are expressed at low levels during infection with the pathogen. In this study, we examined an expression library comprised of approximately 30% of the full ASFV genome. These antigens were selected to include virion or cell surface proteins based on established or predicted cellular locations or known immunogenicity. In addition, virus proteins of other functional classes or expression times were selected. This approach mimicked the profile of proteins expressed during ASFV infection and included a number of highly conserved enzymes. The cloning strategy for expression library construction has varied between random cloning of different-size fragments to the cloning of intact open reading frames (ORFs) if sufficient sequence data are available (21).

Our approach was to use DNA prime and recombinant vaccinia virus boost for delivery of complete or large fragments of ASFV genes to pigs. This dual modality of delivery has been demonstrated to enhance the immune response to the expressed transgenes and to be effective in inducing memory cytotoxic T cell responses as well as inducing antibody responses. The replication-competent modified NYVAC vaccinia virus strain was selected for this purpose since it has been shown to have a good immunogenicity and safety profile (18). To provide flexibility in screening of different antigens, the ASFV genes were individually cloned in both a DNA vector and rVACV and were then delivered in pools to pigs.

The antigens selected included the previously described immunogenic proteins discussed in the introduction in addition to a further 6 antigens which were known to be expressed on the surface of the intracellular mature or extracellular enveloped ASFV particles or on the surface of infected cells. These included KP177R (an early membrane protein), a KP177R-related protein (L10L), and structural proteins B438L, D117L, O61R, and E120R (22). These antigens were selected as potentially important for induction of a protective antibody response or for use as serological markers of infection. An additional 30 ASFV antigens were selected semirandomly to represent genes of different functional classes, including genes for enzymes involved in replication, immune evasion proteins, multigene family members, or proteins of unknown function. In total, 47 whole or partial ASFV genes were screened.

In the first experiment, we screened the immune responses to individual antigens delivered in pools of 22 (sets 1 and 2 [Table 1]) by DNA prime and rVACV boost. The antigens p30 and p72 were included in each pool, but otherwise the pools did not overlap. Cellular immune responses to individual proteins were assayed by stimulation of lymphocytes from immunized pigs with recombinant proteins produced by *in vitro* translation in an E. coli system and detection of the numbers of responding cells by IFN-γ ELISpot assay. By this approach we ranked the antigens according to the relative levels of response induced and consistency of response between different pigs. In both groups of pigs the strongest response in all but one of the pigs was to antigen 127 (CP204L/p30). Other antigens in set 1 which consistently induced high responses included antigens 070 (F317L), 052 (MGF505-4R), 004 (MGF360-11L), 074b (F1055L), 111 (B602L), 128 (CP530R), and 167 (E199L). In set 2, antigens 084 (EP364R), 123 (G1211R), 124 (G1211R), 126a (CP2475L), 133a (NP1450L partial), and 163 (E183L) consistently induced strong responses.

The phenotype of the IFN-γ-producing cells stimulated by antigens was not further characterized. However, it is expected that they would include predominantly CD4^+^ T cells but could also include CD8^+^ T cells if the recombinant antigen was taken up in antigen-presenting cells and processed for presentation of peptides with SLA I. It is known that CP204L/p30 is a highly immunogenic protein and stimulates a strong antibody response during ASFV infection. CP204L/P30 has also been shown to contain epitopes that stimulate CTL responses. The other top-ranked antigens have not previously been identified as proteins against which antibody or cellular immune responses are directed. Antibody responses induced against the individual proteins were tested by ELISA for a subset of antigens selected as known virus or cell surface proteins. The antibody response to p30 protein was highest. Proteins 145 (D117L) and 205 (EP153R) also induced antibody responses more than 2-fold above that seen at day 0. Further investigation is needed to establish if these proteins could be used as novel targets for serological diagnosis. However, ASFV-specific neutralizing antibodies were not induced.

The immunization and challenge experiment demonstrated that pigs immunized with pools of 46 or 47 ASFV antigens by DNA prime and rVACV boost had decreased levels of ASFV genome in blood and some tissues after challenge compared to those in control pigs. We cannot exclude that virus is not cleared earlier but that its distribution to specific tissues is inhibited or altered. Our results suggest that some of the antigens have protective potential. Future experiments will be directed at determining if specific pools of antigens can induce a protective immune response to ASFV challenge. The goal of this work is to develop a safe vaccine strategy for prevention of ASF in pigs.

## MATERIALS AND METHODS

### Construction and testing of expression libraries.

Sequence data of the Georgia 2007/1 ASFV isolate (GenBank accession number FR682468) were used to design primers for amplification of ASFV genes shown in Table 1. The PCR fragments were cloned into the plasmid vector pCMVi-LS (15), which includes a cytomegalovirus promoter for expression in mammalian cells upon DNA vaccination and a signal peptide for cell secretion. The same genes, fused to a C-terminal HA epitope, were inserted into the thymidine kinase locus of modified NYVAC replication-competent vaccinia virus (18) under the control of a vaccinia virus promoter. Expression of a protein of the expected size was confirmed by Western blotting of infected cell extracts and probing with anti-HA antibody (data not shown). The same ASFV genes were also prepared as PCR-generated template DNAs for thioredoxin-tagged protein expression and capture using an E. coli-based *in vitro* transcription translation system (see below).

### Production of DNA templates and antigen capture by *in vitro* transcription translation.

Linear expression elements (LEEs) (23) were used as template DNA for the *in vitro* transcription translation reactions. LEEs were prepared as described previously (16) except using either iProof High-Fidelity (Bio-Rad) or *Pfu* Turbo (Agilent) DNA polymerase plus template purification with the QIAquick PCR purification kit (Qiagen). These LEEs allowed expression of each antigen as a fusion with an N-terminal thioredoxin tag and a C-terminal His_5_ tag.

The *in vitro* transcription translation reactions were done in total volumes of 200 μl. To a Protein LoBind tube (Eppendorf; catalog no. 022431081) was added 50 μl of Dynabeads M-280 tosylactivated magnetic beads (Invitrogen; catalog no. 142.03). The beads were washed three times in 2.4 M ammonium sulfate and 1 M boric acid, pH 9.5 (buffer A), and then coated with antibody overnight at 37°C with shaking at 1,000 rpm (Eppendorf; ThermoMixer) in a combined 30 μl of buffer A and 30 μl of anti-thioredoxin mouse IgG2a monoclonal antibody (GenScript; catalog no. A00180). The supernatant was removed and verified to contain <0.05 mg/ml of IgG as determined by absorbance at 280 nm. The beads were blocked in 2 ml of 0.5% bovine serum albumin in phosphate-buffered saline (PBS; 10 mM Na_2_HPO_4_, 1.76 mM KH_2_PO_4_, 136 mM NaCl, 2.7 mM KCl [pH 7.4]) for 1 h at 37°C and 1,000 rpm, washed three times with PBS, and suspended in PBS using the above-stated volume. A 50-μl volume of these beads was added per well to a 96-well plate (1.2 ml per square well, U bottomed, with lid; ABgene; catalog no. AB-1127), and the supernatant was removed. To the beads was added 100 μl of starter reaction mixture as previously described (16) and containing 1 μg of LEE template DNA. The reaction proceeded as described previously (16), including addition of 100 μl of feed reaction mixture (yielding a total reaction volume of 200 μl), washes in PBS, and storage at −20°C. Antigen yields were determined by phosphorimager analysis following SDS-PAGE (as described previously [16], except with sample heating at 85°C) from parallel reaction mixtures containing [^35^S]methionine (see Fig. S1 in the supplemental material). Twenty-eight of the 47 antigens were obtained in full-length form. The remaining 19 antigens that could not be efficiently generated as full-length products were expressed as fragments that, when combined, encompassed the full-length forms (Fig. S1).

### African swine fever viruses and cells.

The Georgia 2007/1 isolate (24) was grown in primary macrophages from pig bone marrow. Virus stocks for pig inoculation were prepared from a spleen suspension from an infected pig, and titers were determined by limiting dilution using hemadsorption to detect virus-infected cells and calculation by the Spearman-Karber method (25).

### Pig immunization and challenge.

Pigs were either outbred cross-bred Large White and Landrace from a high-health-status farm or inbred Babraham pigs (26) from a herd kept at The Pirbright Institute. Pigs were an average size of 15 kg at the start of experiments. Plasmid DNA was delivered in DNA gold micronanoplex bullets by gene gun (16) by 5 nonoverlapping shots to the pinnae of pigs, at a pressure of 450 lb/in^2^. Plasmid DNA was delivered by a double prime 1 day apart to alternate ears and by a double boost 2 weeks later. CpG oligonucleotide was also bound to the bullets to act as an adjuvant for porcine Toll-like receptors (TLRs) 3 and 4. The total amount of DNA administered was 10 μg per shot. As controls, pigs were vaccinated in the same way with the same dose of DNA in a pool of 3 negative-control plasmids.

Recombinant vaccinia viruses (rVACVs) were delivered by scarification or by using a needleless delivery device at four different sites with a 100-μl total volume at each site. rVACVs were delivered to pigs 4 weeks after the last DNA boost in experiment 1. In experiment 2, pigs were boosted at 3 weeks and 5 weeks with rVACV after the last DNA immunization. Each rVACV was present at 10^8^ PFU for each vaccination. As controls, pigs were vaccinated with a pool of rVACV expressing irrelevant antigens. Pigs were challenged intramuscularly with 10^4^ HAD_50_ of Georgia 2007/1 virus 2 weeks after the last rVACV boost. Pigs were observed for development of clinical signs, and these were scored using the scoring system used previously (27). Blood and tissue samples were collected to measure levels of virus replication.

### Binding of DNA to gold particles.

Target genes from ASFV and negative-control genes (influenza virus hemagglutinin [HA], human α1-antitrypsin [AAT], and HIV envelope gp120) were cloned into the immunization vector pCMVi-LS (15). DNA plasmids were loaded on DNA-gold micronanoplexes as previously described (15, 16), with the following specifications. The amount of DNA per bullet was 5 μg of pooled antigens, divided equally across the antigens, plus 5 μg of CpG adjuvant (Invitrogen; oligonucleotide 5′-ggTGCATCGATGCAGgggggG-3′, where lowercase indicates phosphorothioated nucleotides). Prior to preparation of DNA-nanogold (16), the combined DNA, without CpG, was precipitated in 0.3 M sodium acetate, pH 5.5, and 70% ethanol. The DNA pellet was washed with 70% ethanol, dried for 15 min at room temperature, and dissolved in water.

### Quantitative PCR.

ASFV DNA was detected in blood and tissues using qPCR as described previously (27, 28). The results were determined as genome copies per milliliter of blood or per milligram of tissue.

### Analysis of immune responses against ASFV and against individual recombinant proteins.

Development of T cell immune responses to ASFV was analyzed by interferon gamma (IFN-γ) ELISpot and proliferation assays as described previously (29). The ASFV Georgia isolate used in the assay was grown in porcine bone marrow cells, and 10^5^ HAD_50_ was added per well. Individual thioredoxin-tagged antigens were produced by transcription and translation *in vitro* in an E. coli system and captured by anti-thioredoxin antibody bound to magnetic beads. The beads with antigen attached were suspended in RPMI 1640 medium, and approximately 2.5 μg of *in vitro*-translated protein was added to individual wells in 96-well ELISpot plates. Immune lymphocytes from immunized pigs (6 × 10^5^ cells per well) were added, and incubation was carried out overnight at 37°C before detection of IFN-γ-producing cells. The development of ASFV-specific antibodies was measured using a competition ASFV ELISA (Ingenasa; PPA3 Compac). ELISAs were performed as described previously (13), with the following specifications: per well, 0.1 μg of *in vitro*-translated protein was used; pig serum was used at a 1:500 dilution; well washes were performed in PBS with 0.05% Tween 20 and with the ELISA plate on top of a magnetic rack; and detection was done with TMB (3,3′,5,5′-tetramethylbenzidine) substrate plus HCl and measurement at a wavelength of 450 nm. Total IgG responses used horseradish peroxidase (HRP)-conjugated goat anti-swine IgG (H+L) secondary antibody at a 1:3,000 dilution. Secondary antibodies for IgG1 and IgG2 responses were HRP-conjugated mouse anti-pig IgG1 (clone K139 3C8) and IgG2 (clone K68 Ig2) monoclonal antibodies at a 1:100 dilution.

## Supplementary Material

Supplemental material
