# Supplementary material for: Immunization of Pigs by DNA Prime and Recombinant Vaccinia Virus Boost To Identify and Rank African Swine Fever Virus Immunogenic and Protective Proteins
Source: J Virol. 2018 Mar 28;92(8):e02219-17. doi: 10.1128/JVI.02219-17 (PMC5874426; doi:10.1128/JVI.02219-17)
Supplement: Supplemental material [file supp_92_8_e02219-17__index.html]

Immunization of Pigs by DNA Prime and Recombinant Vaccinia Virus Boost To Identify and Rank African Swine Fever Virus Immunogenic and Protective Proteins — Supplemental material 

# Immunization of Pigs by DNA Prime and Recombinant Vaccinia Virus Boost To Identify and Rank African Swine Fever Virus Immunogenic and Protective Proteins

## Supplemental material

- Supplemental file 1 -

  Fig. S1 (Expression of antigens by *in vitro* transcription translation.)

  PDF, 650K
