# Supplementary material for: Immunization of Pigs by DNA Prime and Recombinant Vaccinia Virus Boost To Identify and Rank African Swine Fever Virus Immunogenic and Protective Proteins
Source: J Virol. 2018 Mar 28;92(8):e02219-17. doi: 10.1128/JVI.02219-17 (PMC5874426; doi:10.1128/JVI.02219-17)
Supplement: Supplemental material [file JVI.02219-17_zjv008183455s1.pdf]

Supplemental Figure 1

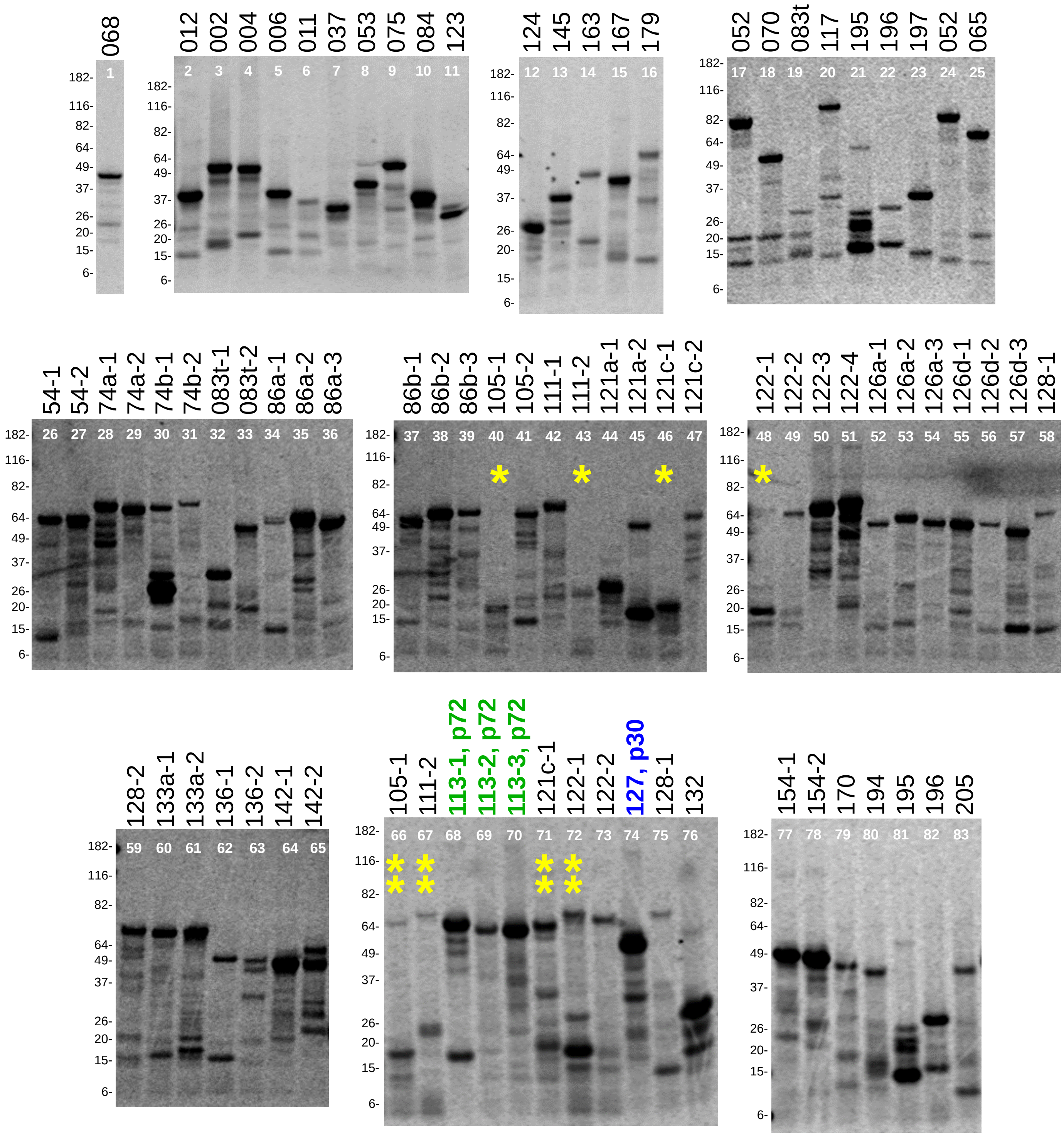

Supplemental Figure 1. Expression of antigens by *in vitro* transcription translation. Shown are phosphorimages following SDS-PAGE from *in vitro* reactions containing ^{35}S-methionine. Each panel originates from an independent gel. For ELISpot and ELISA analyses, antigens were generated from parallel reactions lacking radiolabel. Antigens that could not be generated as full-length products (e.g., 083t, lane 19) were expressed as fragments (e.g., 083t-1 and 083t-2, lanes 32 and 33) that were then combined for immunological analyses. Truncated forms (single asterisk) of 105-1 (lane 40), 111-2 (lane 43), 121c-1 (lane 46) and 122-1 (lane 48) could be expressed in full-length form (double asterisk; lanes 66, 67, 71 and 72, respectively) following optimization of template PCR conditions. Antigen 121a-1 (lane 44) yielded only truncated fragments and so may lack some epitopes. Reproducible expression is seen in replicate reactions for epitopes 052 (lanes 17 and 24), 195 (lanes 21 and 81), 196 (lanes 22 and 82), 122-2 (lanes 49 and 73), and 128-1 (lanes 58 and 75).
